# Surface wave excitations and backflow effect over dense polymer brushes

**DOI:** 10.1038/srep22257

**Published:** 2016-03-15

**Authors:** Sofia Biagi, Lorenzo Rovigatti, Francesco Sciortino, Chaouqi Misbah

**Affiliations:** 1 Université Grenoble Alpes/CNRS UMR 5588, LIPhy, 38041 Grenoble, France; 2 Dipartimento di Fisica, Sapienza-Universitá di Roma, Piazzale A. Moro 5, 00185 Roma, Italy; 3 Faculty of Physics, University of Vienna, Boltzmanngasse 5, A-1090 Vienna, Austria; 4 Istituto Sistemi Complessi (ISC), Via dei Taurini 19, 00185 Roma, Italy

## Abstract

Polymer brushes are being increasingly used to tailor surface physicochemistry for diverse applications such as wetting, adhesion of biological objects, implantable devices and much more. Here we perform Dissipative Particle Dynamics simulations to study the behaviour of dense polymer brushes under flow in a slit-pore channel. We discover that the system displays flow inversion at the brush interface for several disconnected ranges of the imposed flow. We associate such phenomenon to collective polymer dynamics: a wave propagating on the brush surface. The relation between the wavelength, the amplitude and the propagation speed of the flow-generated wave is consistent with the solution of the Stokes equations when an imposed traveling wave is assumed as the boundary condition (the famous Taylor’s swimmer).

Polymer brushes are passive media whose great variety allows for a rich range of applications. Brushes with different mechanical properties can be created by grafting simple polymers, block copolymers or polymer stars to a solid substrate or to an interface between two liquids. They are exploited for colloid stabilisation^1^,^2^,^3^, as lubricant layers^4^,^5^,^6^, as adhesion regulators^7^,^8^ and for biomedical and technological applications. A holdover interest in these systems is motivated by the discovery that the inner surface of various mammalian organs is decorated by densely grafted macromolecules. For example, the lumen of blood vessels is protected by a hundred-nanometers thick polymer brush mainly made of glucose and its compounds. Such a heterogeneous network is called “glycocalyx”^9^,^10^. Research on the glycocalyx dynamics is central for a complete understanding of the blood circulatory system and, hopefully, to shed light on the initial stages of diseases such as thrombosis and atherosclerosis^11^. Understanding glycocalyx is also relevant to the polymeric coatings of lungs, small intestine and uterus^12^,^13^,^14^.

Most theoretical studies have attempted to model polymer brushes as porous media described by Brinkman-type equations^15^ or as elastic media composed of very rigid fibers^16^. Here we report on a numerical investigation of the dynamics of a polymer brush whose features approach the glycocalyx system and for which the external conditions resemble the microcirculation frame^17^. We focus on dense polymer brushes under flow, in which the brush is modelled as a collection of individual polymers. We implement a Dissipative Particle Dynamics (DPD) code^18^,^19^,^20^,^21^ with explicit solvent to numerically analyse the dynamics of a linear flexible homo-disperse polymer brush subdued to a simple liquid parabolic flow in a slit-pore geometry. The coarse-grained DPD procedure applies to both solvent molecules and polymer monomers, offering the possibility to (i) reproduce hydrodynamic interactions while retaining a detailed view of the brush dynamics on the scale of the coarse-grained monomers; (ii) access both the polymer dynamics, influenced by the imposed flow, and the flow field, perturbed by the presence and motion of the brush.

Recent studies of polymer brushes under flow have highlighted an unexpected behaviour of the velocity profile in the vicinity of the brush surface^22^,^23^,^24^. These studies have reported that the velocity field reverses on increasing the flow field and have tentatively associated such result to the peculiar dynamics of the single polymer undergoing a cyclic motion of stretching, elongation and recoiling. Stimulated by these studies we have undertaken a numerical investigation exploring a very large range of imposed flows, aiming at quantifying the conditions under which the physical properties of the brush couple with the hydrodynamic properties of the solvent to produce flow inversion. As presented below, we discover (i) that flow inversion appears in distinct regions of imposed flow values; (ii) that every time flow inversion is observed a surface wave appears, demonstrating that the backflow is strongly associated to a collective (as opposed to single) polymer dynamics; (iii) that the wave properties are consistent with predictions derived by Taylor in his seminal study on pusher microswimmers^25^. Thus, our work presents a new interpretation for the flow inversion phenomenon and establishes a link between two distinct fields: polymer brushes under flow and microswimmers.

## Results

**Figure 1:**
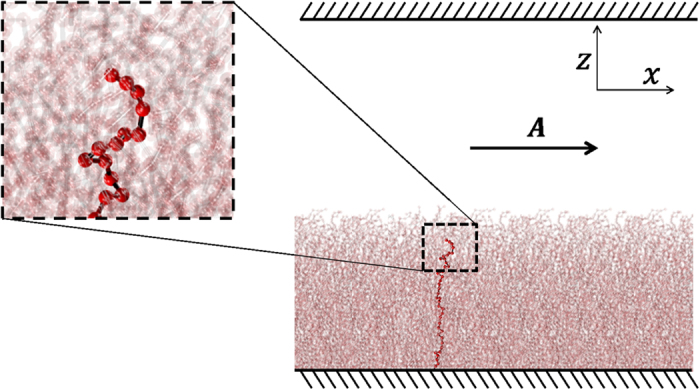
Schematic representation of the investigated slit-pore system. Polymers are grafted at the bottom wall *z* = 0 while the rest of the channel is occupied by solvent particles. An additional sketch is provided with a zoom on a typical chain (highlighted in red). The constant acceleration *A* is applied along the *x* axis to all solvent particles.

A short description of the DPD methodology and of its application to the slit-pore geometry is reported in the Supplementary Information (S.I., Sect.1). Here we briefly describe the system under consideration. We simulate a parallelepiped box of sides *L*_*x*_, *L*_*y*_, *L*_*z*_, with polymers chains composed by 40 (coarse-grained) monomers grafted at *z* = 0 (see Fig. 1). Most of the simulations are carried out in a box of size *L*_*x*_ = 30, *L*_*y*_ = 5 and *L_z_* = 50, in units of *r*_*c*_ (see S. I. for definitions). We remark that we observe the same phenomenology also in considerably bigger boxes (*L*_*x*_ = 360, *L*_*y*_ = 20 and *L*_*z*_ = 150). However, for the sake of computational time, a systematic analysis has been conducted only in the smaller channel. The equilibrium distance between neighbour monomers in a chain is 0.89*r*_*c*_. The grafting density *σ*_*graft*_ ≡ *N*_*ch*_/(*L*_*x*_*L*_*y*_) = 1.5, with *N*_*ch*_ the number of chains composing the brush, corresponds to the condition of dense brush. Periodic boundary conditions are applied along the *x* and *y* directions. In the channel, a flow is imposed by applying a constant acceleration 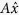 to all solvent particles, resulting in a parabolic velocity profile along the *z* direction. The strength of the velocity is controlled by the value of *A*. In the following, instead of *A*, we will use the so-called Weissenberg number 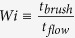, which is a dimensionless number defined as the ratio between the characteristic timescale of the unperturbed brush dynamics (*t*_*brush*_) over the inverse of the averaged shear rate inside the channel (*t*_*flow*_). Specific values for all parameters and their units, as well as a detailed discussion of the *A* dependence of *t*_*flow*_, are reported in the S.I., Sects.1 and 3.

**Figure 2:**
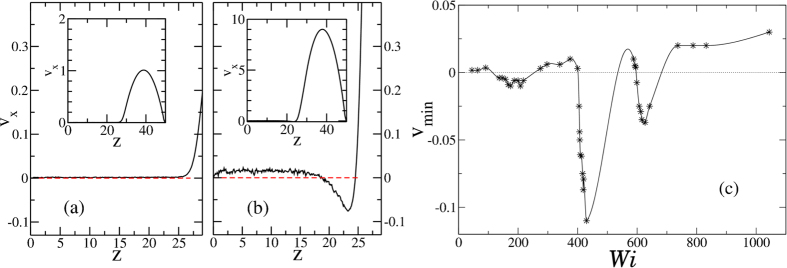
Panels (**a**,**b**) show the velocity profile in the whole channel (insets) and in the region close to the brush (main panels). In both cases, corresponding to (**a**) *Wi* = 45 and (**b**) *Wi* = 418, the profile is parabolic around *v*_*max*_. In (**a**) no backflow is registered, while in (**b**) the flow inversion at *z* ~ *h* is evident. Panel (**c**) reports the minimum value *v*_*min*_ of the *v*_*x*_(*z*) profile for different *Wi* (symbols). The line is a guide for the eye. It is possible to recognize three regions for which *v*_*min*_ assumes a negative value, signalling the presence of a backward flow.

The velocity profile *v*_*x*_(*z*), calculated by averaging the *x*-component of the velocity for each solvent particle as a function of its position *z*, is shown in Fig. 2(a,b) for two *Wi* values, providing two typical examples. As expected, the functional shape of *v*_*x*_(*z*) in the region around the largest velocity *v*_*max*_ is well represented by a parabolic function with the expected amplitude. In all cases, the parabolic function predicts that *v*_*x*_ vanishes when *z* approaches the brush height *h*, suggesting that, for all studied *Wi*, the presence of a dense polymer brush restricts the pore by an amount equivalent to *h*. The velocity profile in the region *z* < *h*, *i.e.* the region densely occupied by the brush, is of particular interest. In some cases, *e.g.* the one presented in Fig. 2(a), the fluid velocity inside the brush is small, consistent with a picture in which the hydrodynamic interactions are effectively screened by the presence of the polymer layer. In other cases, as shown in Fig. 2(b), the velocity profile exhibits a flow inversion at the interface between the brush and the bulk, *i.e.* around *z* ~ *h*, as previously documented for an analogous system^24^ as well as when the solvent is replaced by a polymer melt^22^,^23^. In these previous studies, the onset of flow inversion was associated to the shear rate exceeding a certain threshold^24^. Instead, we find the existence of at least three different windows of flow intensities in which a backflow is observed. In between these regions the velocity profile at *z* ~ *h* remains positive, resembling the one in Fig. 2(a). Figure 2(c) shows the minimum value *v*_*min*_ of the profile *v*_*x*_(*z*) near the surface of the polymer brush, as a function of *Wi*. By this definition, flow inversion is observed when *v*_*min*_ < 0. The identified three backflow regions fall around *Wi* ~ 200, 400, 600, respectively.

In refs 22, 23, 24 it was argued that the backflow is a consequence of the dynamics of single grafted chains, which undergo a recursive, imperfect cycle composed of: tilting, elongation and recoiling. Indeed, an elongated chain (and thus exposed to flow) is dragged by the shear stress along the flow direction and then recoiled back by entropic forces. By examining the polymer trajectories we confirm the presence of such recursive motion (see movie in the S.I., Sect.4) for all *Wi* values, but such a cyclic motion is always observed, regardless of the presence of the backflow. Hence, the single-polymer motion alone is not enough to explain the onset of flow inversion.

**Figure 3:**
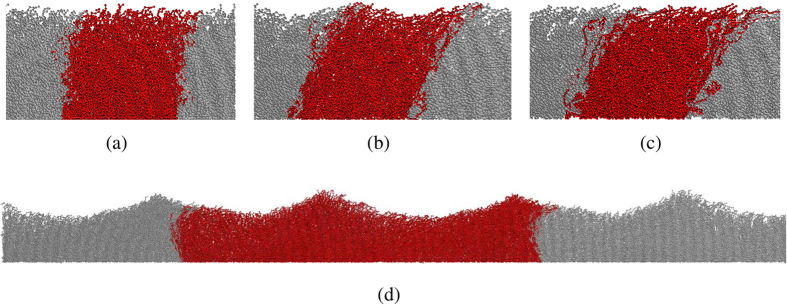
Sketches of the whole brush, corresponding to (**a**) *Wi* = 91, (**b**) *Wi* = 418 and (**c**) *Wi* = 588. In (**a**) the brush surface is basically flat, in (**b**) the surface is modulated by a travelling wave with *λ* ~ *L*_*x*_ = 30, *ν* = 0.077 and *b* = 1.18 while in (**c**) the brush filaments tilt but no surface waves are present. In snapshot (**d**) a larger studied systems (*L*_*x*_ = 180, *L*_*y*_ = 5 and *L*_*z*_ = 150) exhibits a surface wave with multiple crests of wavelength *L*_*x*_/2 ≈ 90 (*Wi* = 340). We show the central box (in red) and the two nearest replicas (in grey) to highlight the periodic nature of the waves.

**Figure 4:**
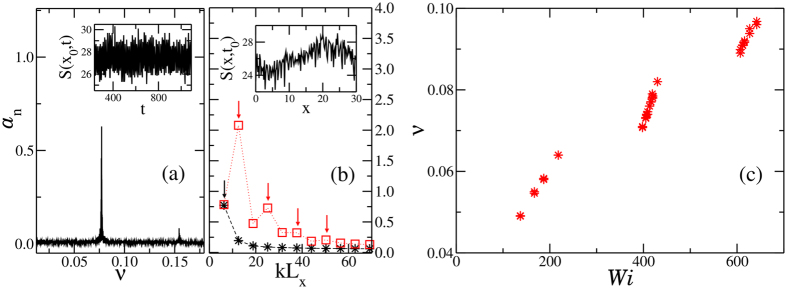
Fourier power-spectra: (**a**) in the frequency domain *ν* for the signal *S*(*x*_0_, *t*), (**b**) in the *kL_x_* domain, where *k* = 2*π*/*λ* is the wavenumber, for the signal *S*(*x*, *t*_0_) (red squares indicate the spectrum in a bigger system *L*_*x*_ = 180, *L*_*y*_ = 5 and *L*_*z*_ = 150). The arrows highlight the position of the peaks. In both panels the first and second harmonics are present. The two insets show typical signals in real space. Panel (**c**) shows the characteristic frequency of the waves, which are found to be monotonically increasing functions of *Wi*.

Investigating space and time correlations among different polymers, we discover a wave travelling over the brush surface in the same direction as the imposed flow. The wave arises *if and only if* a backflow is present, suggesting a strong association between the surface wave and the capability of the brush to produce an inversion of the flow velocity around *z* ~ *h*. A visual inspection of the brush and of its dynamics nicely shows the collective behaviour of the polymers. Figure 3(a–c) show typical snapshots of the system at low flow (no wave), intermediate flow (a surface wave appears) and at higher flows where waves are absent, respectively. Videos of the time dependence of the brush are available in the S.I., Sect.4. We find that this travelling wave has a clear spatial periodicity of the order of the simulation box and a non-negligible amplitude. We characterize the surface wave by evaluating its frequency *ν*, wavelength *λ* and oscillation amplitude *b*. In order to do so we define the brush surface *S*(*x*, *t*) as the position of the farthest monomer from the grafting wall occupying at time *t* the coordinate *x*, averaged over all *y*. The power-spectrum of the time Fourier transform of *S*(*x*_0_, *t*) (for an arbitrary *x*_0_ value, inset of Fig. 4(a)) displays, in all cases in which a flow inversion is observed, a sharp peak at a given frequency, as exemplified in Fig. 4(a). We associate the position of this peak with the characteristic wave frequency *ν*. In some cases a much less intense peak is also observed at 2*ν*. Fourier transforming the signal *S*(*x*, *t*_0_) in real space for an arbitrary *t*_0_ and averaging over all configurations provides a quantification of the wavelength *λ*. As shown in Fig. 4(b), for all cases in which a flow inversion is present we find, as in the time domain, dominant contributions from the longest *λ* that can propagate in a periodic system of size *L*_*x*_ and from its second harmonic, *i.e.*, from wavenumbers *k* equal to 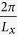 and 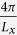 respectively. Figure 4(c) shows the *Wi* dependence of *ν* for systems with the same *L*_*x*_ (*e.g.* same *λ*). In each of the three disconnected regions of *Wi* values where flow inversion is present, *ν* increases approximatively linearly with *Wi*. Finally, we define the wave amplitude *b* as the standard deviation of *S*(*x*, *t*) and the wave average height as 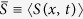.

We note that wave propagation is observed also in systems of different *L*_*x*_. We find that on increasing the length *L*_*x*_ and the width *L*_*z*_ of the channel, multiple wave crests can be displayed (see Fig. 3(d)). This suggests that the finite size of the simulation box exerts a cutting on the density of states, making it possible to observe only waves with wavenumbers which are integer multiples of 2*π*/*L*_*x*_. In addition, we find that the values of *Wi* at which we observe oscillations depend on *L*_*x*_ and *L*_*z*_. We ascribe this dependence to the appearance of new characteristic time scales, for example the relaxation time of brush compression produced by the pressure drop Δ*P* = *ρAL*_*x*_, which are not taken into account in the definition of the Weissenberg number. Further details on finite size effects are reported in the S.I., Sect.5.

We interpret the co-presence of flow inversion and surface waves in light of the work done by Taylor on the mobility of microswimmers^25^. In his seminal paper, dating back to 1951, Taylor investigates the motion of a viscous fluid near a sheet under which sinusoidal waves are propagated with velocity *U*. If the ratio between the wave amplitude *b* and the wavelength *λ* is not too small, the sheet moves with a velocity *V*_*Taylor*_ in the opposite direction with respect to *U*. The relation between the sheet self-propelling velocity and the travelling wave features reads:


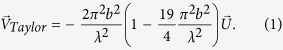


**Figure 5:**
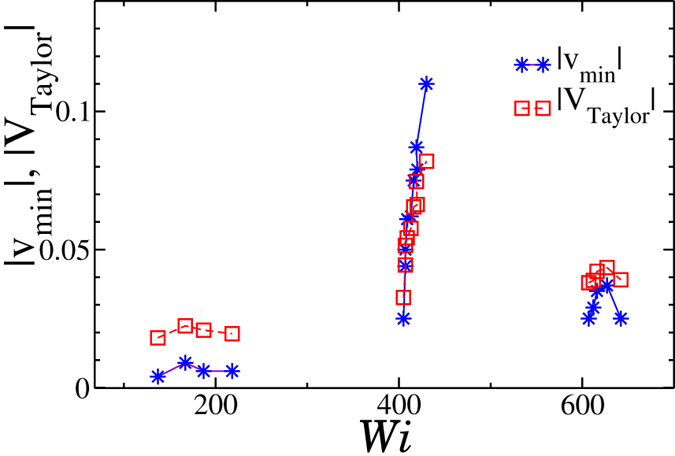
The mapping between the absolute value of the velocity profile minimum |*v*_*min*_| (stars) and the velocity *V*_*Taylor*_ obtained by plugging in Eq. (1) the wave amplitude *b*, the wavelength *λ* and the wave velocity *U* = *λν* evaluated for the brush surface wave at the given *Wi*.

We associate the two-dimensional sheet with the brush surface and *U* with the velocity *λν* of the brush surface wave. Since the polymer brush is grafted to the wall and cannot translate but it affects the fluid close to its surface, we identify *V*_*Taylor*_ with the velocity of the solvent at the brush interface *v*_*min*_. Therefore we exploit Eq. (1) to build a precise relation between the parameters entering in the wave phenomenon and the parameters controlling the flow inversion. Figure 5 shows, for each set of *b*, *λ* and *U* values associated to a specific *Wi* value giving flow inversion, the comparison between |*V*_*Taylor*_| (calculated from Eq. (1)) and |*v*_*min*_|. The trends associated with the behaviour of |*v*_*min*_| are perfectly recovered by the self-propelling velocity of a Taylor’s micro-swimmer, suggesting that the elastic brush can be considered as an anchored micro-swimmer, unable to move but able to propel the surrounding liquid.

As a last consideration, we investigate possible origins of the surface wave. Our results suggest that the wave propagation arises from hydrodynamics interactions synchronising polymers, in a sort of metachronal motion. A “ metachronal wave” is known to develop in ciliated systems, namely flat surfaces covered by a regular matrix made of equally spaced flexible filaments^26^,^27^. Exposed to a flow, filaments do not interact directly, but thanks to hydrodynamics interactions, after some transient time, they reach a synchronized state with a regular phase shift between subsequent filament rows. However, in those cases the matrix is composed by active matter elements and the sequence of configurations they assume is assigned *a priori*. On the contrary, polymer brushes are passive media. Different approaches have been developed to model the wave formation on passive media such as crop canopies (because of flowing wind) or aquatic vegetations (because of water flow), but they require also the inertial term of Navier-Stokes equations to account for the instability initiating the surface modulation^28^,^29^. However, we can consider the brush to be an elastic medium. Therefore, the presence of oscillations of finite amplitude suggests that a possible resonance effect may arise. Indeed, the pressure load exerted by a viscous fluid flow can induce an elastic compressible system to develop an instability called *flutter*, *i.e.* the appearance of spontaneous oscillations at preferential frequencies. As elucidated in ref. 30, in the limit of weak hydrodynamic coupling and small amplitudes, the system always develops a fluttering instability for Weissenberg numbers larger than a certain critical value. Therefore, the phenomenology differs from the one discussed here, which exhibits three well-separated instability regions. We speculate that such difference is due to the strong non-linear behaviour exhibited by the polymer brush, which constitutes a much more complex system than the simply elastic one considered in ref. 30. Specifically, we find that, in contrast with the latter, the properties of the brush have a significant dependence on *Wi*: as a function of the imposed flow, the brush stiffens and its height decreases (see S.I.). Those effects are not included in the theoretical framework introduced by Mandre and Mahadevan^30^. The development of an extension of their theoretical approach to the more complicated case of a system with flow-dependent properties is under way.

## Discussion

In summary, we have studied the behavior of dense polymer brushes under parabolic flow by means of numerical simulations. Increasing *Wi* we have detected three ranges of imposed flow for which a velocity inversion appears at the interface between the brush and the rest of the channel. We have discovered a well-characterized surface traveling wave, which appears in all cases of backflow and it is not present in the other ones, providing a novel picture of the flow-inversion phenomenon, which is associated to a collective motion of the whole brush rather than to the single chain dynamics. We have also observed a striking and unexpected similarity in the relation between the wave velocity and the backflow velocity for the case of flow in passive polymer brushes and for the case of active microswimmers^25^.

## Additional Information

**How to cite this article**: Biagi, S. *et al.* Surface wave excitations and backflow effect over dense polymer brushes. *Sci. Rep.* **6**, 22257; doi: 10.1038/srep22257 (2016).

## Supplementary Material

Supplementary Information

Supplementary Video 1

Supplementary Video 2

Supplementary Video 3

Supplementary Video 4

Supplementary Video 5

## References

[b1] Pincus P. Colloid stabilization with grafted polyelectrolytes. Macromolecules 24, 2912–2919 (1991).

[b2] Lo Verso F., Egorov S. A., Milchev A. & Binder K. Spherical polymer brushes under good solvent conditions: Molecular dynamics results compared to density functional theory. J. Chem. Phys. 133, 184901 (2010). 21073226 10.1063/1.3494902

[b3] Jaquet B. *et al.* Stabilization of polymer colloid dispersions with ph-sensitive poly-acrylic acid brushes. Colloid Polym. Sci. 291, 1659–1667 (2013).

[b4] Kobayashi M., Tanaka H., Minn M., Sugimura J. & Takahara A. Interferometry study of aqueous lubrication on the surface of polyelectrolyte brush. ACS Appl. Mater. Interfaces 6, 20365–20371 (2014). 25340883 10.1021/am505906h

[b5] Klein J., Kumacheva E., Mahalu D., Perahia D. & Fetters L. Reduction of frictional forces between solid surfaces bearing polymer brushes. Nature 370, 634–636 (1994).

[b6] Chen M., Briscoe W., Armes S. & Klein J. Lubrication by biomimetic surface layers at physiological pressures. Science 323, 1698–1701 (2009). 19325108 10.1126/science.1169399

[b7] Ramakrishna S. N., Espinosa-Marzal R. M., Naik V. V., Nalam P. C. & Spencer N. D. Adhesion and friction properties of polymer brushes on rough surfaces: A gradient approach. Langmuir 29, 15251–15259 (2013). 24266663 10.1021/la402847z

[b8] Landherr L., Cohen C., Agarwal P. & Archer L. Interfacial friction and adhesion of polymer brushes. Langmuir 27, 9387–9395 (2011). 21696203 10.1021/la201396m

[b9] Weinbaum S., Tarbell J. M. & Damiano E. R. The structure and function of the endothelial glycocalyx layer. Annu. Rev. Biomed. Eng. 9, 121–167 (2007). 17373886 10.1146/annurev.bioeng.9.060906.151959

[b10] Reitsma S., Slaaf D., Vink H., van Zandvoort M. & oude Egbrink M. The endothelial glycocalyx: composition, functions, and visualization. Pflugers Arch. 454 (2007). 10.1007/s00424-007-0212-8 PMC1915585 17256154

[b11] Nieuwdorp M. *et al.* The endothelial glycocalyx: a potential barrier between health and vascular disease. Curr. Opin. Lipidol. 16, 507 (2005). 16148534 10.1097/01.mol.0000181325.08926.9c

[b12] Schmidt E. P. *et al.* The pulmonary endothelial glycocalyx regulates neutrophil adhesion and lung injury during experimental sepsis. Nat. Med. 18, 1217–1223 (2012). 22820644 10.1038/nm.2843 PMC3723751

[b13] Murphy C. & Turner V. Glycocalyx carbohydrates of uterine epithelial cells increase during early pregnancy in the rat. J. Anat. 17, 109–115 (1991). 1769885 PMC1260418

[b14] Button B. *et al.* A periciliary brush promotes the lung health by separating the mucus layer from airway epithelia. Science 337, 937–941 (2012). 22923574 10.1126/science.1223012 PMC3633213

[b15] Secomb T. W., Hsu R. & Pries A. R. Motion of red blood cells in a capillary with an endothelial surface layer: effect of flow velocity. Am. J. Physiol. Heart. Circ. Physiol. 281, H629–H636 (2001). 11454566 10.1152/ajpheart.2001.281.2.H629

[b16] Weinbaum S., Zhang X., Han Y., Vink H. & Cowin S. C. Mechanotransduction and flow across the endothelial glycocalyx. Proc. Natl. Acad. Sci. 100, 7988–7995 (2003). 12810946 10.1073/pnas.1332808100 PMC164700

[b17] Lanotte L., Guido S., Misbah C., Peyla P. & Bureau L. Flow reduction in microchannels coated with a polymer brush. Langmuir 28, 13758–13764 (2012). 22935030 10.1021/la302171a

[b18] Hoogerbrugge P. & Koelman J. M. V. A. Simulating microscopic hydrodynamic phenomena with dissipative particle dynamics. Europhys. Lett. 19, 155 (1992).

[b19] Groot R. D. & Warren P. B. Dissipative particle dynamics: Bridging the gap between atomistic and mesoscopic simulation. J. Chem. Phys. 107, 4423 (1997).

[b20] Español P. & Warren P. B. Statistical mechanics of dissipative particle dynamics. Europhys. Lett. 30, 191 (1995).

[b21] Ilnytskyi J., Sokolowski S. & Patsahan T. Dissipative particle dynamics study of solvent mediated transitions in pores decorated with tethered polymer brushes in the form of stripes. Condens. Matter Phys. 16, 13606 (2013). 10.1063/1.3592562 21639473

[b22] Léonforte F., Servantie J., Pastorino C. & Müller M. Molecular transport and flow past hard and soft surfaces: computer simulation of model systems. J. Phys. Condens. Matter 23, 184105 (2011). 21508476 10.1088/0953-8984/23/18/184105

[b23] Müller M. & Pastorino C. Cyclic motion and inversion of surface flow direction in a dense polymer brush under shear. Europhys. Lett.)

[b24] Deng M., Li X., Liang H., Caswell B. & Karniadakis G. E. Simulation and modelling of slip flow over surfaces grafted with polymer brushes and glycocalyx fibres. J. Fluid Mech. 711, 192–211 (2012). 10.1017/jfm.2012.387 PMC3864822 24353347

[b25] Taylor G. Analysis of the swimming of microscopic organisms. Proc. R. Soc. A 209, 447–461 (1951).

[b26] Elgeti J. & Gompper G. Emergence of metachronal waves in cilia arrays. Proc. Natl. Acad. Sci. 110, 4470–4475 (2013). 23487771 10.1073/pnas.1218869110 PMC3607033

[b27] Blake J. R. A model for the micro-structure in ciliated organisms. J. Fluid Mech. 55, 1–23 (1972).

[b28] Gosselin F. & de Langre E. Destabilising effects of plant flexibility in air and aquatic vegetation canopy flows. Eur. J. Mech. B-Fluid. 28, 271–282 (2009).

[b29] Babenko V. V., Chun H. H. & Lee I. Boundary Layer Flow over Elastic Surfaces (Butterworth-Heinemann, Oxford, 2012).

[b30] Mandre S. & Mahadevan L. A generalized theory of viscous and inviscid flutter. Proc. R. Soc. A (2009).

