# Supplementary material for: Surface wave excitations and backflow effect over dense polymer brushes
Source: Sci Rep. 2016 Mar 15;6:22257. doi: 10.1038/srep22257 (PMC4792148; doi:10.1038/srep22257)
Supplement: Supplementary Information [file srep22257-s1.pdf]

Supplementary Information to Surface wave excitations and backflow effect over dense polymer brushes

Sofia Biagi, Lorenzo Rovigatti, Francesco Sciortino, and Chaouqi Misbah

1 DPD methodology

DPD is a coarse-grained Molecular Dynamics method. It was introduced by Hoogerbrugge and Koelman in 1992 [1] to simulate isothermal Navier-Stokes equations and it has been recently recognized that it can be successfully applied to any mesoscopic scale [2]. In the spirit of DPD, the simulated particle does not correspond to a single molecule, but rather to a significant large *cluster* of them. Each of the N point-like particles evolves in time according to Newton's equations

$$m_i \dot{\vec{v}}_i = \sum_{j \neq i}^{N} \vec{F}_{ij} = \sum_{j \neq i}^{N} (\vec{F}_{ij}^C + \vec{F}_{ij}^D + \vec{F}_{ij}^S) \tag{1}$$

that we solve by using the velocity Verlet algorithm [3]. Observables are then calculated as averaged all over time configurations. The force $\vec{F}_{ij}$ of Eq. 1 on each particle has three contributions [4] resulting from the coarse-graining procedure: a *conservative* component $\vec{F}_{ij}^C$, a *dissipative* component $\vec{F}_{ij}^D$ and a *stochastic* one $\vec{F}_{ij}^S$. All these forces are pair-wise, to guarantee momentum conservation, and limited to a cut-off radius r_c.

The conservative soft-core repulsive force has the following expression:

$$\vec{F}_{ij}^C = \begin{cases} a_{\alpha,\beta} \left(1 - \frac{r_{ij}}{r_c}\right) \hat{r}_{ij} & r_{ij} \leq r_c, \\ 0 & r_{ij} > r_c \end{cases} \tag{2}$$

with $\vec{r}_{ij} = \vec{r}_i - \vec{r}_j$ is the vector distance between the i-th and j-th particle, $r_{ij} = |\vec{r}_{ij}|$ and $\hat{r}_{ij} = \vec{r}_{ij}/r_{ij}$. Such repulsive potential models the averaged fast microscopic length and time scales and was first derived by coarse-graining particles interacting via a Lennard-Jones potential [5]. The indexes α, β indicates the particle type (solvent or polymer, in our case). The constant $a_{\alpha,\beta}$ measures the force between two completely overlapping particles and it is casted from the compressibility of the modeled fluid.

The two other forces account for the loss of details in the coarse-graining procedure. The dissipative force has the form:

$$\vec{F}_{ij}^D = -\gamma w^D(r_{ij})(\hat{r}_{ij} \cdot \vec{v}_{ij})\hat{r}_{ij}, \tag{3}$$

where the standard choice in literature for the "weight function" $w^D(r_{ij})$ is:

$$w^D(r_{ij}) = \begin{cases} \left(1 - \frac{r_{ij}}{r_c}\right)^2 \hat{r}_{ij} & r_{ij} \leq r_c, \\ 0 & r_{ij} > r_c. \end{cases} \tag{4}$$

Equation (3) introduces a friction among particles proportional to the relative velocity $\vec{v}_{ij} = \vec{v}_i - \vec{v}_j$ Thermal fluctuations are added via the stochastic force:

$$\vec{F}_{ij}^S = \sigma w^S(r_{ij})\theta_{ij}(\Delta t)^{-\frac{1}{2}}\hat{r}_{ij} \tag{5}$$

where σ is a constant (related to temperature), $w^S(r_{ij})$ is a weight function and θ_{ij} is a random number extracted from a gaussian distribution with zero average and indipendent in time and among particle pairs: $\langle\theta_{ij}(t)\theta_{lm}(t')\rangle = (\delta_{il}\delta_{jm}+\delta_{im}\delta_{jl})\delta(t-t')$. Momentum conservation requires $\theta_{ij} = \theta_{ji}$.

Two additional constraints, $w^D(r_{ij}) = [w^R(r_{ij})]^2$ and $\gamma = \frac{\sigma^2}{2k_BT}$, guarantee that the probability distribution respects the statistics of the NVT ensamble [6].

To mimic the brush chains we add a finite extensible nonlinear elastic potential (FENE) [7]

$$\vec{F}_{ij}^{fene} = -2kR^2\frac{r_{ij} - r_{eq}}{R^2 - (r_{ij} - r_{eq})^2}\hat{r}_{ij} \qquad r_{ij} - r_{eq} < R \tag{6}$$

which account for the neighbour particle connectivity. In Eq. (6), r_{eq} is the equilibrium distance between neighbour monomers and R is the maximum allowed extensibility. Grafted points are randomly chosen from a uniform distribution and located on a flat surface. Note that polymers are non-ideal since they interact via an excluded volume potential.

1.1 DPD and physical units

We chose the DPD units such as $r_c = 1$, $m_i = 1$, $t_{DPD} = 1$ and $k_BT = 1$. Following [4], we fix the solvent-solvent interaction parameter a_{SS} relating it to the adimensional compressibility of water κ_T^{-1}. A comparison between the equation of state of a simulated DPD system and the experimental data ($\kappa_T^{-1}(water) = 16$ at room temperature) suggests $a_{SS} = 75k_BT/\rho = 25$. We assume that the polymer-polymer interaction parameter has the same value (e.g. $a_{SS} = a_{PP}$), while we select a smaller value for the solvent-polymer parameter $a_{SP} = 20$ (*good solvent* conditions). The noise amplitude is fixed to $\sigma = 3$. For the FENE potential, finally, we use $r_{eq} = 0.86$, $R = 1$ and $k = 50$.

In DPD the choice for the physical units depends on the level of coarse-graining is desired. Our reference system is the endothelial glycocalyx, therefore we fix the physical lengthscale l_{phys} relating the spacing between different filaments of the glycocalyx network, $d_{glyco} = 20nm$ [8] with the average distance between anchor points of our brush, $d_{graft} = \sqrt{1/\sigma_{graft}} = 0.82$:

$$l_{phys} = d_{glyco}/d_{graft} = 24 \cdot 10^{-9}m.$$

We underline that with this choice also the brush thickness is in the range of endothelial glycocalyx ($100nm \div 1000nm$ [9]).

As for the physical mass scale m_{phys} and physical time scale t_{phys}, we exploit the comparison between viscosities and between energies. The viscosity of water η_{phys} is $\eta_{phys} = 10^{-3}Pa\ s$ at $300K$ and the DPD viscosity η_{DPD} in a bare channel, estimated from the slit-pore velocity profile relation $\eta = \rho A(L_z/2)^2/2v_{max}$, where v_{max} is the maximum velocity (see Sect. 1.2 for definitions of the other parameters), corresponds in our case to $\eta_{DPD} = 0.84$. The physical energy scale is defined as k_BT_{phys}, with $T_{phys} = 300K$. Thus we can write down two relations

$$m_{phys} = \frac{\eta_{phys}}{\eta_{DPD}}t_{phys}l_{phys},$$

$$t_{phys} = \frac{l_{phys}}{v_{phys}} = \frac{l_{phys}}{\sqrt{\frac{3k_B T_{phys}}{m_{phys}}}},$$

from which we extract

$$t_{phys} = \frac{l_{phys}^3}{3k_B T_{phys}} \frac{\eta_{phys}}{\eta_{DPD}} = 1.8 \cdot 10^{-6} s,$$

$$m_{phys} = \frac{\eta_{phys}}{\eta_{DPD}} t_{phys} l_{phys} = 5.1 \cdot 10^{-17} Kg.$$

1.2 The system geometry

We simulate a parallelepiped box of sides $L_x = 30$, $L_y = 5$ and $L_z = 50$. On the x- and y- axes we impose periodic boundary conditions, while at $z = 0$ and $z = L_z$ two impenetrable parallel walls of infinite mass are set. At the wall we use the so-called "bounce back reflection"conditions [10] in which all the velocity components are reversed:

$$\vec{v} \to -\vec{v} \quad \text{at} \quad z = 0, L_z. \tag{9}$$

Such conditions assure no-slip boundaries. The integration time step is $\Delta t = 0.02$. We set the density $\rho = N/(L_x L_y L_z) = 3$ [4], therefore $N = 22500$. We fix the number of monomers per chain $n = 40$ and the grafting density $\sigma_{graft} = 1.5$, defined as $\sigma_{graft} = N_{ch}/(L_x L_y)$, with N_{ch} the number of chains composing the brush. For the sake of ease and computational time, we attach polymers only at the bottom wall.

In order to produce a parabolic velocity profile inside the channel (e.g. along the z direction) a constant acceleration $\vec{A} = A\hat{x}$ is applied to all fluid particles:

$$m_i \dot{\vec{v}}_i^{fluid} = \vec{F}_{ij}^{fluid} + m_i \vec{A}. \tag{10}$$

In turn, these fluid particle exchange momentum with the chains, dragging them. Different values of A allow us to probe different dynamic regimes.

2 Brush equilibrium properties

Polymer brushes immersed in a liquid at equilibrium (i.e. in the absence of flow) have been thoroughly investigated in the past [11, 12, 13, 14]. Here we recall that the brush equilibrium conformation results from the balance between configurational entropy, that tends to make chains visit the whole available space, and excluded volume interactions, avoiding contact between monomers. The brush can be properly described by its profile $\rho(z)$, indicating the probability distributions of finding a monomer at distance z from the grafting wall. We show in Fig. 1 $\rho(z)$ at fixed $\sigma_{graft} = 1.5$ for several chain lengths ($n = 20, 25, 30, 36, 40, 45$). In line with previous theoretical results [12] and numerical studies [13, 15], the higher the volume fraction, the more step-like the density profile is.

A definition of the brush height h in term of the first moment $< z >$ of the density profile depends on the shape of $\rho(z)$. Assuming that each chain is completely elongated so that free ends are all at the maximum distance from the grafting wall, the profile $\rho(z)$ is step-like (*Alexander model*) and the brush height can be defined as $h = 2 < z >$. A less rigid theory (*self-consistent field theory*) hypothesizes that monomers of a same chain are distributed as a random walk, therefore the free end position ranges uniformely over the whole brush thickness. In such a case the profile is parabolic, $\rho(z) = \rho(0)(1 - z^2/h^2)$, and the brush height h is $h = \frac{8}{3} < z >$. For

the simulations discussed in the manuscript we select $n = 40$, so that the evaluated $\rho(z)$ is in between the step and the parabolic shapes (see Fig. 1). In the following, we define h following the definition of a step distribution, e.g.

$$h = 2\frac{\int_0^\infty z\rho(z)dz}{\int_0^\infty \rho(z)dz}. \tag{11}$$

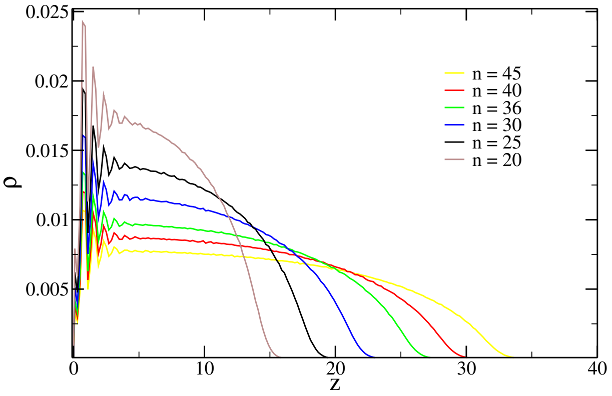

Figure 1: Probability distributions of polymer brushes at equilibrium: fixed the grafting density, $\sigma_{graft} = 1.5$, we varied the degree of polymerization n. The case $n = 40$ corresponds to an almost step-like profile $\rho(z)$.

2.1 Brush compression properties

From simulations at equilibrium ($A = 0$) in which the brush is compressed by a flat surface kept at a fixed position S_{fix} we have estimated the Young's modulus E as $E = \dfrac{P}{\Delta S/S_0}$. In this expression, $\Delta S/S_0 = (S_{fix} - S_0)/S_0$ is the compression of the brush relative to the value S_0 indicating the brush surface without any imposed confinement and P is the pressure exerted by polymers on the pressing apparatus. In Fig. 2 we observe that E is increasing with the compression. We now notice that flow produces a compression of the brush (see Fig. 3). Equating the compression imposed at equilibrium with the one produced by the applied flow (shaded areas in Fig. 2) we conclude that the brush elastic response is modified by a change in Wi.

3 The Weissenberg number

Instead of using A as a measure of the flow intensity we prefer to introduce an adimensional quantity, the Weissenberg number $Wi := t_{brush}/t_{flow}$, where t_{brush} should be a characteristic structural time of the unperturbed brush and t_{flow} should provide an estimate of the typical time scale associated to the flow. We evaluate t_{brush} as the relaxation time obtained by the decay of the autocorrelation function, averaged over all distinct polymers, of the end-to-end vector amplitude $R_{ee} = |\vec{r}_0 - \vec{r}_n|$, where $\vec{r}_n$ and $\vec{r}_0$ are the position vectors of, respectively, the last particle and the anchor of one chain inside the brush. According to such definition, the relaxation time t_{brush} assumes a dependency on the grafting density and on the degree of polymerization, thus representing a time scale of the whole brush rather than an isolated single-chain property.

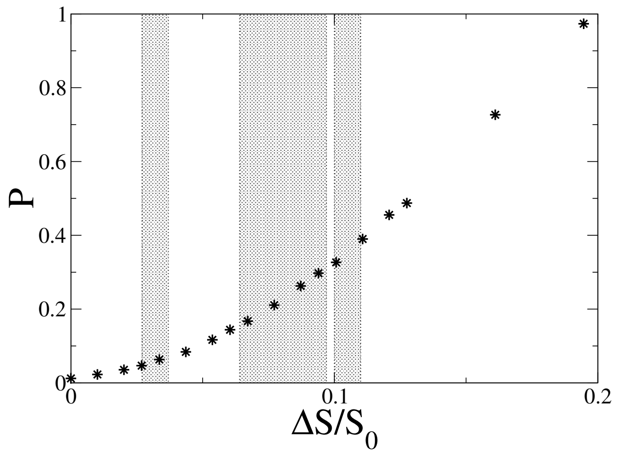

Figure 2: The brush elastic response stored in the Young's modulus E. The modulus is defined as the coefficient between the relative brush compression and the applied pressure needed to produce it. The shaded areas indicate the ranges of brush surface compression corresponding to the three regions of flow inversion.

The R_{ee} auto-correlation function can be well represented by a stretched exponential decay $R_{ee} \sim e^{-(t/t_o)^\beta}$, where t_o is the characteristic time scale and β the so-called stretching exponent. Thus $t_{brush} = \Gamma[1/\beta]t_o/\beta$. For $\sigma_{graft} = 1.5$ and $n = 40$ we obtain $t_{brush} = 1746$. Regarding t_{flow}, we choose the inverse of the averaged shear rate $\dot{\gamma}$ calculated as the ratio between the maximum flow velocity v_{max} in the channel and the corresponding z-coordinate R. Since our brush under flow experiences compression (see Fig. 3) and polymers are set only at the bottom wall, both v_{max} and R depend on the imposed acceleration A.

In table Tab. 1 we list the correspondences between each value of A and its Wi.

4 Videos

We show in movies SI1 and SI2 the dynamics of a single chain inside the brush for two different Wi, one giving flow inversion (movie SI1) and the other with no flow inversion (movie SI2): the recursive motion is constituted by an alternation of elongation-stretching-recoiling in both cases, thus it cannot be used as sufficient explanation for the backflow phenomenon.

The motion of the whole brush under flow has been recorded in movies SI3 and SI4. In the first case no collective motion is detectable and no backward flow is observed in the velocity profile, while in the second case there is concurrence of a surface wave and of flow inversion.

5 Finite size effect

We have investigated the influence of finite size effect increasing the channel length L_x at fixed acceleration A: the wave persists. For a larger channel, $30 < L_x < 40$, the wave increases in wavelength ($\lambda = L_x$) and decreases in frequency, as shown in Fig. 4, such that the propagation speed keeps constant. The wave amplitude decreases and, in accordance with the Taylor's relation (see Letter, Eq. (1)), v_{min} decreases too.

Another test has been attempted increasing, up till the double, both L_x and L_z ($L_x = 60$

Conversion $A \leftrightarrow Wi$

A	Wi	A	Wi	A	Wi	A	Wi
0.005	45	0.022	208	0.047	409	0.064	612
0.007	64	0.023	218	0.048	413	0.065	616
0.01	91	0.028	275	0.05	418	0.066	627
0.015	137	0.03	297	0.051	420	0.067	642
0.016	147	0.034	340	0.055	430	0.07	737
0.017	158	0.037	375	0.0565	588	0.075	788
0.018	167	0.04	400	0.575	593	0.08	832
0.019	175	0.043	405	0.058	595	0.1	1044
0.02	187	0.045	406.7	0.06	599		
0.021	200	0.046	407.4	0.063	607		

Table 1: Tables of conversions between the constant acceleration A, imposed on each solvent particle to obtain a parabolic velocity profile, and the estimated a-dimensional Weissenberg number Wi.

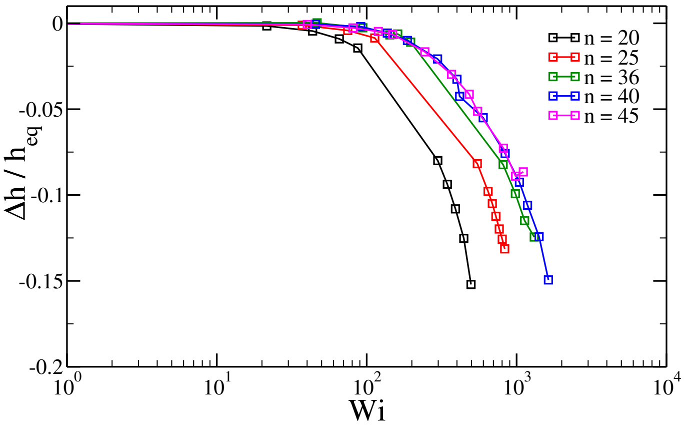

Figure 3: The brush height h relative to the equilibrium value h_{eq} as function of Wi. The brush height is defined in Sec. 2. For $Wi < 100$ the height keeps basically constant, while for $Wi > 100$ the flow has the effect to compress the brush.

and $L_z = 100$). For such a system we found again, for $Wi = 464$, a surface wave, with double amplitude and double wavelength (see movie SI5). However, investigating channels up to $L_x = 360$, $L_y = 20$ and $L_z = 150$, we found that the amplitude of the surface wave does not keep on growing upon increasing L_x: in fact, its value seems to reach a plateau. We interpret this non-linear behaviour as a consequence of the finite extensibility of the polymers, e.g. due to the higher and higher energy required to significantly compress and stretch the brush.

References

[1] P.J. Hoogerbrugge and J. M. V. A. Koelman. Simulating Microscopic Hydrodynamic Phenomena with Dissipative Particle Dynamics. *Europhysics Letters*, 19:155, 1992.

[2] Rudolf M Füchslin, Harold Fellermann, Anders Eriksson, and Hans-Joachim Ziock. Coarse

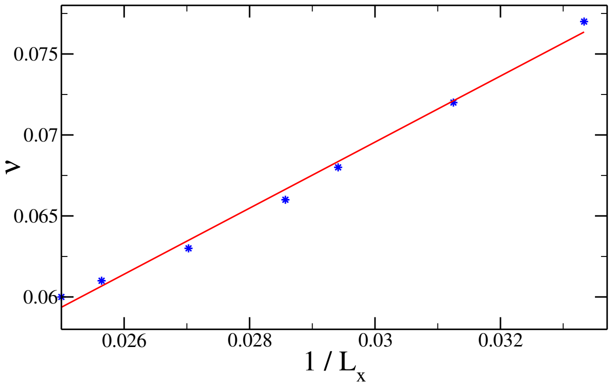

Figure 4: Increasing the channel length L_x, $30 < L_x < 40$, at fixed acceleration $A = 0.05$ the wave increases in wavelength ($\lambda = L_x$) and decreases in frequency such that the propagation speed keeps constant.

graining and scaling in dissipative particle dynamics. *The Journal of chemical physics*, 130(21):214102, June 2009.

[3] D. Frenkel and B. Smit. *Understanding Molecular Simulation: From Algorithms to Applications*. Computational science series. Elsevier Science, 2001.

[4] Robert D. Groot and Patrick B. Warren. Dissipative particle dynamics: Bridging the gap between atomistic and mesoscopic simulation. *The Journal of Chemical Physics*, 107(11):4423, 1997.

[5] Bruce M. Forrest and Ulrich W. Suter. Accelerated equilibration of polymer melts by time-coarse-graining. *The Journal of Chemical Physics*, 102(18):7256, 1995.

[6] P. Español and Patrick B. Warren. Statistical Mechanics of Dissipative Particle Dynamics. *Europhysics Letters*, 30:191, 1995.

[7] G. S. Grest and K. Kremer. Molecular dynamics simulation for polymers in the presence of a heat bath. *Physical Review A*, 33:3628–3631, May 1986.

[8] Sheldon Weinbaum, John M. Tarbell, and Edward R. Damiano. The structure and function of the endothelial glycocalyx layer. *Annual Review of Biomedical Engineering*, 9:121–167, 2007.

[9] B.M. Fu and J.M. Tarbell. Mechano-sensing and transduction by endothelial surface glycocalyx: composition, structure, and function. *Wiley Interdiscip Rev Syst Biol Med*, 5:381–390, 2013.

[10] M. Revenga, I. Zúñiga, and P. Español. Boundary conditions in dissipative particle dynamics. *Computer Physics Communications*, 121-122:309–311, September 1999.

[11] S. T. Milner, T. A. Witten, and M. E. Cates. A parabolic density profile for grafted polymers. *EPL (Europhysics Letters)*, 5:413, March 1988.

[12] S. T. Milner. Polymer Brushes. *Science*, 251:905–914, 1991.

[13] Kurt Binder, Torsten Kreer, and Andrey Milchev. Polymer brushes under flow and in other out-of-equilibrium conditions. *Soft Matter*, 7(16):7159, 2011.

[14] Jean Louis Barrat. A possible mechanism for swelling of polymer brushes under shear. *Macromolecules*, 25(2):832–834, 1992.

[15] C. Pastorino, K. Binder, T. Kreer, and M. Müller. Static and dynamic properties of the interface between a polymer brush and a melt of identical chains. *The Journal of Chemical Physics*, 124(6), 2006.
